# Recurrence Is a Noticeable Cause of Rifampicin-Resistant *Mycobacterium tuberculosis* in the Elderly Population in Jiangxi, China

**DOI:** 10.3389/fpubh.2019.00182

**Published:** 2019-07-19

**Authors:** Qiang Chen, Linfeng Peng, Guangchu Xiong, Yiping Peng, Dong Luo, Lijin Zou, Kaisen Chen

**Affiliations:** ^1^Department of Clinical Laboratory, The First Affiliated Hospital of Nanchang University, Nanchang, China; ^2^Department of Respiratory, The First Affiliated Hospital of Nanchang University, Nanchang, China; ^3^Department of Clinical Laboratory, Jiangxi Chest Hospital, Nanchang, China; ^4^Department of Burns, The First Affiliated Hospital of Nanchang University, Nanchang, China

**Keywords:** tuberculosis, recurrence, rifampicin-resistant, elderly patients, China

## Abstract

**Setting:** Rifampicin-resistant tuberculosis (RR-TB) in elderly people in Jiangxi Province, China.

**Objective:** To investigate the incidence of RR-TB and risk factors in elderly people within a hospital setting in China.

**Design:** Retrospective cohort study.

**Methods:** A comparative study was performed to analyze RR-TB and rifampicin-susceptible TB (RS-TB). The 15-locus mycobacterial interspersed repetitive unit-variable number of tandem repeats (MIRU-VNTR) method was used to distinguish between relapse and reinfection.

**Results:** Twenty-three recurrent cases occurred in 151 elderly patients with RR-TB, and 24 recurrent cases occurred in 466 elderly patients with RS-TB during this period. TB recurrence was significantly different in the RR-TB and RS-TB groups (OR = 0.35, 95% CI: 0.14–0.88; χ^2^ = 5.28, *P* = 0.03). Comparing the risk factors for RR-TB and RS-TB, we found that educational level, age, and pulmonary cavity were inextricably linked to RR-TB in elderly patients. Furthermore, pulmonary cavity, HIV status, and alcohol consumption were associated with recurrence in elderly RR-TB patients.

**Conclusions:** Recurrence is an important source of RR-TB in the elderly population. It is necessary to promptly treat tuberculosis patients, prevent the spread of AIDS, and reduce alcohol intake to control recurrent RR-TB in the elderly population.

## Introduction

Tuberculosis (TB) has remained a serious public health concern in China and is one of the top 10 causes of death and the leading cause of death due to a single infectious agent. The World Health Organization (WHO) estimated that there were 10.0 million new cases of TB, including 0.56 million rifampicin-resistant TB (RR-TB) cases, in 2017, and of these, 82% were multidrug-resistant TB (MDR-TB) (1). Owing to the low success of treatment and longer treatment period, RR-TB patients bear greater spiritual and financial burdens, especially elderly patients who have significantly reduced physical function, owing to comorbidities such as diabetes, hypertension, and rheumatism (2, 3). Identifying the cause of RR-TB is important for its accurate and timely management.

In 2017, China became the country with the largest elderly population in the world, with 240.9 million people aged 60 years and above, accounting for 17.3% of the total population according to the data from the Chinese National Bureau of Statistics. It is manifest that there are many underlying diseases and increasing poverty in the elderly population due to their disability and decreasing ability to perform labor as well as the partial deficiency of the social security system. As a result, some geriatric TB patients have become a growing social problem (4, 5). The underdeveloped economic level and problems associated with an aging population mean that the rapidly increasing incidence of TB in Jiangxi Province is not an optimistic situation (6). It is urgent to know the epidemiological characteristics and infectious patterns in elderly TB patients to support better TB control and prevention.

To determine the prevalence of TB in the elderly population and the characteristics of these elderly patients, especially elderly RR-TB patients, we collected clinical and sociodemographic information from January 2008 to December 2016 in Jiangxi Chest Hospital. Through a comparative study, we identified some risk factors for RR-TB, and we also found that recurrence was a significant factor responsible for RR-TB in the elderly population.

## Study Population and Methods

### Study Population and *Mycobacterium tuberculosis* Isolates

This retrospective study was performed at the Jiangxi Chest Hospital from January 1, 2008, to December 31, 2016, and all data were obtained from the hospital information database. The Jiangxi Chest Hospital is located in Nanchang, an important city in southeast China. Approximately 12,000 patients with pulmonary TB are treated in this hospital every year, coming from all parts of Jiangxi Province. Pulmonary TB patients were recruited during this period. Three sputa samples at different time points (spot, early, and night) from each patient who was suspected of having pulmonary TB were collected for acid-fast bacilli (AFB) testing using the Ziehl Neelsen (ZN) method and Löwenstein–Jensen culturing. Thiophene carboxylic acid hydrazide (TCH) and *p*-nitrobenzoic acid (PNB) were used to differentiate between *M. tuberculosis* complex (MTBC) and non-tuberculosis mycobacteria (NTM). The study was approved by the ethics committee of the First Affiliated Hospital of Nanchang University, Nanchang, China (approval number: 2014036). At the same time, this study was also approved by the Jiangxi Chest Hospital Institutional Review Board (IRB). All personal data were kept confidential. Written informed consent was obtained from each participant prior to the study. We confirmed that all adopted methods were conducted in accordance with the relevant guidelines and regulations in China.

### Drug Susceptibility Testing

For all *M. tuberculosis* isolates collected, drug susceptibility testing (DST) was routinely performed using BACTEC MGIT 960 (BD) to measure susceptibility to eight types of anti-TB drugs according to the MGIT 960 operating manual (7). These drugs included isoniazid (INH) (0.2 μg/ml), rifampin (RIF) (40.0 μg/ml), streptomycin (SM) (4.0 μg/ml), ethambutol (EMB) (2.0 μg/ml), ofloxacin (OFL) (1.0 μg/ml), kanamycin (KAN) (30.0 μg/ml), amikacin (AMK) (40.0 μg/ml), and capreomycin (CMP) (40.0 μg/ml). Quality control was routinely performed using the reference strain H37Rv (ATCC27294). The results were interpreted according to the relevant published literature (8).

### Data Collection and Definitions

During the study period, all information on 3,264 pulmonary TB culture-positive patients at the Jiangxi Chest hospital was obtained. The patients' data were collected, including sex, age, marital status, history of diabetes, TB treatment history, educational levels, presence of pulmonary cavities, HIV status, smoking habits, sputum smear results, and place of residence. The term “elderly TB patients” refers to patients who were no <60 years old, and the term “young TB patients” refers to patients who were <60 years old and more than 18 years old when they were diagnosed with TB at the first episode. Recurrent patents in this study met the criteria previously reported in the literature (9). Essentially, there are two aspects to the criteria for recurrence: (1) completion of the standard anti-TB treatment process and (2) more than two episodes of diagnosed TB (within a minimum time interval of 12 months based on the date of the end of treatment for the first episode). Non-recurrent TB cases are defined as TB patients who test positive for the first time. Relapse, known as endogenous reactivation, is caused by the same strain that caused the first episode of TB. Alcohol consumption refers to the daily consumption of more than 10 g of alcohol, and heavy alcohol consumption refers to a daily consumption of more than 40 g of alcohol. The place of residence refers to the place where the household registration is located, which is usually divided into rural residences and urban residences.

### DNA Extraction and Sequencing

Bacterial genomic DNA was obtained from the isolates by the boiled lysis method. All RIF-resistant strains (MTB isolates from the first episode) were included in this study. A 442-bp oligonucleotide fragment of the *rpoB* gene was amplified and sequenced (including an 81-bp core RIF resistance determining region) [454-FLX Sequencer (Roche), Sangon Bio Co., Shanghai, China]. The primer sequences and PCR conditions were based on previously reported studies (10, 11). The primers were synthesized by Sangon Bio Co., (Shanghai, China). The PCR mixtures were prepared using 2 × *Taq* MasterMix (Tiangen Co., Beijing, China). Sequencing data were assembled and analyzed with Mega5.04 software.

### Genotyping Method

Fifteen-locus MIRU-VNTR amplification was performed to genotype the recurrent RIF-resistant strains following the protocol described in our previous report (12). In brief, all MIRU-VNTRs were amplified by normal PCR, and then the PCR products were separated by electrophoresis on 1.5% agarose gels, and the size analysis of the PCR fragments was performed with the Gel Image Analysis System (LiuYi Co., Beijing, China). The numbers of repetitions of various MIRU-VNTR loci were determined by comparison with the reference strain H37Rv. A reinfection case was defined by the occurrence of strains with different MIRU-VNTR patterns between the first and second TB episodes; a relapse case was defined by the occurrence of strains with the same MIRU-VNTR patterns (9).

### Statistical Analysis

Chi-square tests or Fisher's exact tests were used to compare the proportions of patients in each subgroup (such as RR-TB or RS-TB patients; recurrent or non-recurrent TB patients). Odds ratios (ORs) and 95% confidence intervals (95% CIs) were calculated in the univariate analyses. All statistical procedures were performed with SPSS version 17.0 software (SPSS Inc., Chicago, IL, USA). Values of *P* < 0.05 were considered statistically significant.

## Results

### Recurrence Is an Important Risk Factor for Elderly RR-TB Patients

**Figure 1:**
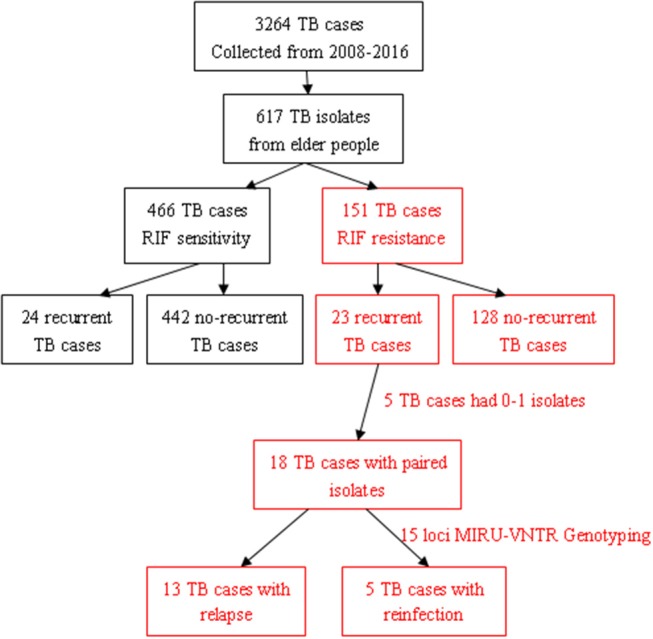
Flow diagram of tuberculosis (TB) cases in this study.

In total, 3,264 pulmonary TB patients visited the Jiangxi Chest Hospital during the indicated period, and 18.90% (617/3,264) of those patients were elderly people. Among them, 24.47% (151/617) had RR-TB, including 23 with recurrent TB and 128 with non-recurrent TB (Figure 1). Among the 23 patients with recurrent TB, 3 died, 19 had one recurrence, and 1 had two or more recurrent episodes during this study period. Furthermore, 75.53% (466/617) of the patients had RIF-susceptible TB, including 24 with recurrent TB and 442 with non-recurrent TB. The results showed that the recurrent status played a more important role in elderly RR-TB patients than in elderly RS-TB patients (OR = 0. 35, 95% CI: 0.14–0.88; *P* = 0.03).

### Clinical Characteristics of Elderly RR-TB Patients

When examining the clinical characteristics of elderly RR-TB patients, we primarily compared them with those of RIF-susceptible elderly patients. The results indicated that educational level, age, and the presence of pulmonary cavities were important risk factors in elderly RR-TB patients. However, factors such as sex, HIV status, alcohol consumption, smear positivity, place of residence, and career had no influence on the appearance of RIF-resistant or RIF-susceptible TB (Table 1). To further understand the characteristics of elderly RR-TB patients, we also compared different clinical and social demographic characteristics between patients with recurrent and non-recurrent TB. Overall, there was a significantly higher proportion of patients with recurrent TB among the HIV-positive patients, indicating that HIV status was an important risk factor for TB recurrence (OR = 5.80, 95% CI: 2.13–15.79; *P* = 0.01). Furthermore, alcohol consumption and the presence of pulmonary cavities also played vital roles in recurrence (OR = 0. 28, 95% CI: 0.11–0.71; *P* = 0.02), particularly in rural men. In contrast, educational level, age, sex, a history of diabetes, smear positivity, and place of residence had no influence on the percentage of patients who relapsed (Table 2).

**Table 1:** Demographic and clinical characteristics of RIF-resistant and RIF-susceptible elderly TB cases in this study.

**Characteristics**	**RIF-resistant cases *n* = 151 (%)**	**RIF-susceptible cases *n* = 466 (%)**	**Total *n* = 617 (%)**	**OR 95% CI**	***P*-value**
**Educational level**					
Primary school	133 (88.08)	332 (71.24)	465 (75.36)	Ref	
Middle or high school	10 (6.62)	85 (18.24)	95 (15.40)	3.41 1.72–6.76	0.001
College or higher	8 (5.30)	49 (10.51)	57 (9.24)	2.45 1.13–5.32	0.02
**Age (years)**					
60–69	42 (27.81)	91 (19.53)	133 (21.56)	Ref	
70–79	66 (43.71)	288 (61.80)	354 (57.37)	2.01 1.28–3.17	0.003
≥80	43 (28.48)	87 (18.67)	130 (21.07)	0.93 0.56–1.57	0.90
**Sex**					
Male	107 (70.86)	342 (73.39)	449 (72.77)	1.130.76–1.70	0.60
Female	44 (29.14)	124 (26.61)	168 (27.23)	Ref	
**Diabetes**					
Yes	43 (28.48)	138 (29.61)	181 (29.33)	Ref	
No	108 (71.52)	328 (70.39)	436 (70.67)	0.95 0.63–1.42	0.84
**Pulmonary cavity**					
Yes	55 (36.42)	119 (25.54)	174 (28.20)	Ref	
No	96 (63.58)	347 (74.46)	443 (71.80)	1.67 1.13–2.47	0.01
**HIV status**					
Negative	136 (90.07)	434 (93.13)	570 (92.38)	Ref	
Positive	15 (9.93)	32 (6.87)	47 (7.62)	0.67 0.35–1.27	0.22
**Alcohol consumption**					
No	92 (60.93)	265 (65.63)	357 (57.86)	Ref	
Yes	59 (39.07)	201 (34.37)	260 (42.14)	1.18 0.81–1.72	0.40
**Smear positive**					
No	51 (21.74)	158 (33.91)	209 (33.87)	Ref	
Yes	100 (78.26)	308 (66.09)	408 (66.13)	0.99 0.67–1.46	1.00
**Residents**					
Urban residents	26 (13.04)	82 (17.60)	108 (17.50)	Ref	
Rural residents	125 (86.96)	384 (82.40)	509 (82.50)	0.97 0.60–1.58	1.00

**Table 2:** Demographic and clinical characteristics of RIF-resistant elderly TB cases in this study.

**Characteristics**	**Recurrent cases *n* = 23 (%)**	**Non-recurrent cases *n* = 128 (%)**	**Total *n* = 151 (%)**	**OR 95% CI**	***P*-value**
**Educational level**					
Primary school	18 (78.26)	115 (89.84)	133 (88.08)	Ref	
Middle or high school	3 (13.04)	7 (5.47)	10 (6.62)	0.370.09–1.54	0.16
College or higher	2 (8.70)	6 (4.69)	8 (5.30)	0.470.09–2.51	0.32
**Age (years)**					
60–69	8 (34.78)	34 (26.56)	42 (27.81)	0.850.31–2.33	0.80
70–79	11 (47.83)	55 (42.97)	66 (43.71)	Ref	
≥80	4 (17.39)	39 (30.47)	43 (28.48)	1.950.58–6.58	0.40
**Sex**					
Female	6 (26.09)	38 (29.69)	44 (29.14)	Ref	
Male	17 (73.91)	90 (70.31)	107 (70.86)	0.840.31–2.28	0.81
**Diabetes**					
Yes	12 (52.17)	46 (35.94)	58 (38.41)	Ref	
No	11 (47.83)	82 (64.06)	93 (61.59)	1.950.80–4.76	0.17
**Pulmonary cavity**					
Yes	17 (73.91)	42 (32.81)	59 (39.07)	Ref	
No	6 (26.09)	86 (67.19)	92 (60.93)	5.802.13–15.79	0.001
**HIV status**					
Negative	17 (72.91)	119 (92.97)	136 (90.07)	Ref	
Positive	6 (26.09)	9 (7.03)	15 (9.93)	4.671.48–14.76	0.01
**Alcohol consumption**					
No	8 (34.78)	84 (65.63)	92 (60.93)	Ref	
Yes	15 (65.22)	44 (34.37)	59 (39.07)	0.280.11–0.71	0.02
**Smear positive**					
No	5 (21.74)	46 (35.94)	51 (33.77)	Ref	
Yes	18 (78.26)	82 (64.06)	100 (66.23)	0.500.17–1.42	0.24
**Residents**					
Urban residents	3 (13.04)	23 (17.97)	26 (17.22)	Ref	
Rural residents	20 (86.96)	105 (82.03)	125 (82.78)	0.690.19–2.50	0.77

### Molecular Characteristics of RR-TB Strains in Elderly Patients

Except for 8 wild-type strains, the remaining RIF-resistant strains harbored mutations within the 81-bp RIF resistance determining region (RRDR). Altogether, 94.7% (143/151) of the RR-TB isolates harbored mutations in 10 residues in RRDR. The most frequently mutated rpoB was Ser531 (91/151, 60.3%), with 87 Ser531Leu and 4 Ser531Phe, of which 15 appeared in patients with recurrent TB and 76 appeared in patients with non-recurrent TB. The second most frequently mutated point was residue His526, with 4 His526Asp, 13 His526Pro, and 4 His526Leu. All five double mutants showed mutations in the core region RRDR. There was no significant difference between patients with recurrent and non-recurrent TB (*P* > 0.05) (Table 3).

**Table 3:** Distribution of mutations in rpoB gene among 151 RIF-resistant MTB from elderly patients in this study.

**Locus**	**Change**		**No. (%) of isolates**	
	**Nucleotide**	**Amino acid**	**No. (%) of recurrent cases (*n* = 23)**	**No. (%) of non-recurrent cases (*n* = 128)**
rpoB531	TCG 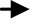 TTG	Ser 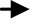 Leu	14 (60.87)	73 (57.03)
	TCG 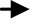 TTT	Ser 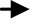 Phe	1 (4.35)	3 (2.34)
rpoB526	CAC 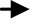 GAC	His 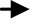 Asp	1 (4.35)	3 (2.34)
	CAC 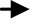 CCC	His 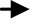 Pro	1 (4.35)	12 (9.38)
	CAC 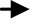 CTC	His 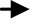 Leu	1 (4.35)	3 (2.34)
rpoB516	GAC 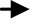 GTC	Asp 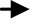 Val	1 (4.35)	6 (4.69)
rpoB516/511	GAC 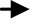 GGC CTG 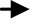 CCG	Asp 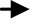 Gly Leu 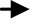 Pro	0 (0.00)	2 (1.56)
rpoB533	CTG 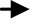 CCG	Leu 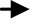 Pro	2 (8.70)	4 (3.12)
rpoB533/518	CTG 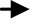 CCG ATG 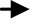 GTG	Leu 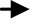 Pro Met 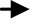 Val	0 (0.00)	3 (2.34)
rpoB513	CAA 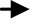 AA	Gln 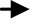 Lys	1 (4.35)	8 (6.25)
rpoB519	AAC 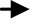 AAG	Asn 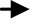 Lys	0 (0.00)	1 (0.01)
Wild type	None	None	1 (4.35)	7 (5.47)

### Drug-Resistance Profiles in RR-TB Strains in Elderly Patients

**Figure 2:**
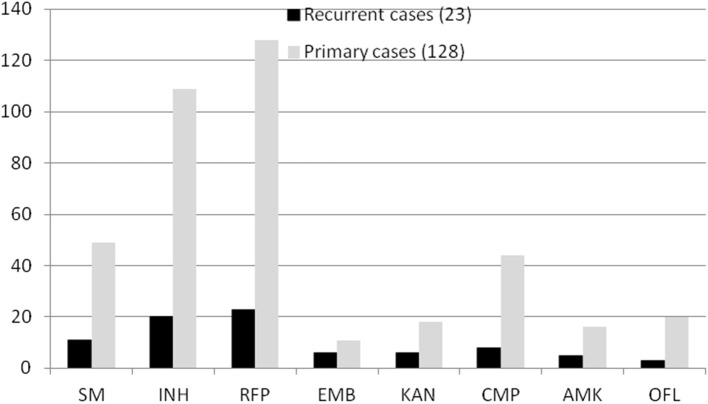
Comparison pattern of drug resistance in 151 RR-TB strains.

In total, 151 RR-TB strains were included in our study. Some strains carried other drug-resistant patterns. For example, in all 23 patients with recurrent TB, 11 strains were resistant to SM, 20 were resistant to INH, 6 were resistant to EMB, 3 were resistant to KAN, 8 were resistant to CMP, 5 were resistant to AMK, and 3 were resistant to OFL. Correspondingly, in all 128 patients with non-recurrent TB, 49 strains were resistant to SM, 109 were resistant to INH, 11 were resistant to EMB, 18 were resistant to KAN, 44 were resistant to CMP, 16 were resistant to AMK, and 20 were resistant to OFL (Figure 2). However, there was no significant difference between patients with recurrent and non-recurrent TB (*P* > 0.05).

### Relapse Was More Common in Elderly RR-TB Patients

During this study period, 16 of 23 patients with recurrent TB acquired second strains, and the MIRU-VNTR results showed that 13 had identical genotypes and 3 acquired different genotypes. As a result, we considered that relapse was the major reason (13/16, 81.25%) for recurrence in those patients. When comparing the effects of different factors on relapse or reinfection, we found no statistically significant differences based on gender, age, educational level, HIV status, alcohol consumption, smear positivity, the presence of pulmonary cavities, or career (data not shown).

## Discussion

To better understand the situation of elderly TB patients in Jiangxi Province, we performed a detailed investigation of culture-confirmed patients from January 1, 2008, to December 31, 2016, in a tertiary TB care hospital. We found 617 elderly TB patients among the overall 3,264 patients, which showed that elderly people still constitute a large proportion of the patients with TB (13). According to the local demographic data, the proportions of elderly with TB were relatively high in all age groups, which was consistent with the results of previous studies (1, 14). Furthermore, we found 151 RR-TB patients among the 617 elderly TB patients, and the isolation ratio was relatively high. Considering that most RR-TB patients have MDR-TB (14, 15), the incidence of MDR-TB appears to be very high among elderly people in Jiangxi Province (5). According to The International Union Against Tuberculosis and Lung Disease, RR-TB should be treated similarly to MDR-TB (16).

To determine the mutation characteristics of the *rpoB* gene among these recurrent RR-TB strains, we sequenced the RRDR of the *rpoB* gene. We found that RIF resistance occurred primarily in the RRDR region of the *rpoB* gene, consistent with the findings of a previous report (17). We also compared it with TB strains isolated from patients with non-recurrent TB, and the results indicated that there were no obvious differences, which was consistent with some reports in the literature (18, 19).

To prioritize the strategies for RR-TB control in the elderly population, it is necessary to know the risk factors associated with RR-TB in elderly patients. We compared RR-TB patients with RS-TB patients, and the results indicated that people with higher educational levels were less likely to acquire RR-TB. Compared with RS-TB, RR-TB was more likely to originate from recurrence and treatment failure (20–23); people with higher educational levels are more likely to have a highly structured life, making it easier to adhere accurately to a drug regimen (24, 25). The presence of pulmonary cavities was another risk factor; RR-TB is an independent predictor of acquiring MDR-TB, and the presence of pulmonary cavities is an independent risk factor for MDR-TB (26).

Recurrence was a significant source of RR-TB among elderly patients (*P* < 0.05), so we sought to determine the risk factors for recurrence by comparing patients with recurrent TB with those with non-recurrent TB. Recurrence usually occurs if there is universal incorrect usage of rifamycin to treat primary TB, according to the WHO guidelines (14). Furthermore, HIV positivity also had an important effect on RR-TB recurrence in this study; while that result was similar to the finding reported by Unis et al. (27), Guerra-Assuncǎo et al. (28) found that HIV positivity primarily affected reinfection rather than relapse. Our MIRU-VNTR data showed that 50% (3/6) of HIV-positive patients with recurrent TB had been reinfected, and 50% had experienced relapse, which is inconsistent with results reported in the literature (28, 29). The reason may be the small sample size. Alcohol consumption was another risk factor, especially long-term heavy drinking in male patients (data not shown). Patients who engage in long-term heavy drinking usually live less regularly scheduled lives and do not adhere to the anti-TB drug regimen as required; furthermore, long-term drinking also impairs immune function (30). A long-term multiregional study has also confirmed a positive correlation between alcohol consumption and TB (31). Although some studies (32–34) found that diabetes had an important effect on TB occurrence, our study focused on elderly RR-TB patients; as a result, there was no significant difference between those with and without diabetes.

Our report had several limitations. First, the collected information was incomplete. The collection of TB patients did not originate from all TB units in the province but rather from the only comprehensive tertiary hospital; as a result, the information pertaining to some patients was not acquired if they did not visit the same hospital the next time. Furthermore, patients were not enrolled if they were only suspected of having relapse but did not have evidence of the same strain twice, which led to the underestimation of relapse. Furthermore, the interpretation of relapse and reinfection in elderly RR-TB patients should be considered with caution due to the limited number of specimens from patients with recurrent TB. However, because the Jiangxi Province Chest Hospital is the only tertiary TB hospital in Jiangxi Province, almost all patients with refractory disease, treatment failure, and recurrent TB seek care in this hospital; as a result, the study results have a high degree of credibility.

## Conclusion

Our study demonstrated that recurrence is a very important factor for RR-TB in elderly patients in Jiangxi Province. It is necessary to administer more stringent standardized treatment to and conduct regular TB examinations in those patients.

## Data Availability

The raw data supporting the conclusions of this manuscript will be made available by the authors, without undue reservation, to any qualified researcher.

## Author Contributions

All authors listed have made a substantial, direct and intellectual contribution to the work, and approved it for publication.

### Conflict of Interest Statement

The authors declare that the research was conducted in the absence of any commercial or financial relationships that could be construed as a potential conflict of interest.
